# Elastography Study of Hamstring Behaviors during Passive Stretching

**DOI:** 10.1371/journal.pone.0139272

**Published:** 2015-09-29

**Authors:** Guillaume Le Sant, Filiz Ates, Jean-Louis Brasseur, Antoine Nordez

**Affiliations:** 1 Laboratory ‘Movement, Interactions, Performance’ (EA 4334), Faculty of Sports Sciences, University of Nantes, Nantes, France; 2 School of Physiotherapy (IFM3R), Nantes, France; 3 Pitié Salpêtrière Hospital, radiology service, AP-HP, Paris, France; University of Zaragoza, SPAIN

## Abstract

**Introduction:**

The mechanical properties of hamstring muscles are usually inferred from global passive torque/angle relationships, in combination with adjoining tissues crossing the joint investigated. Shear modulus measurement provides an estimate of changes in muscle-tendon stiffness and passive tension. This study aimed to assess the passive individual behavior of each hamstring muscle in different stretching positions using shear wave elastography.

**Methods/Results:**

The muscle shear modulus of each hamstring muscle was measured during a standardized slow passive knee extension (PKE, 80% of maximal range of motion) on eighteen healthy male volunteers. Firstly, we assessed the reliability of the measurements. Results were good for *semitendinosus* (*ST*, CV: 8.9%-13.4%), *semimembranosus* (*SM*, CV: 10.3%-11.2%) and *biceps femoris long-head* (*BF-lh*, CV: 8.6%-13.3%), but not for *biceps femoris short-head* (*BF-sh*, CV: 20.3%-44.9%). Secondly, we investigated each reliable muscle in three stretch positions: 70°, 90° and 110° of hip flexion. The results showed different values of shear modulus for the same amount of perceived stretch, with the highest measurements in the high-flexed hip situation. Moreover, individual muscles displayed different values, with values increasing or *BF-lh*, *SM* and *ST*, respectively. The inter-subject variability was 35.3% for *ST*, 27.4% for *SM* and 30.2% for *BF-lh*.

**Conclusion:**

This study showed that the hip needs to be high-flexed to efficiently tension the hamstrings, and reports a higher muscle-tendon stress tolerance at 110° of hip angle. In addition muscles have different passive behaviors, and future works will clarify if it can be linked with rate of injury.

## Introduction

Flexibility is believed to be a fundamental for daily living and well-being [1, 2] in addition to be controversially associated with the risk of sport injuries.[3–5] Therefore, stretching is popular among health therapists and athletic trainers.[6] The classical method used to study hamstrings flexibility *in vivo* focuses on the maximum range of motion (ROM) measurement and resistance to motion (ie, passive torque, expressed by angular variation) offered by the musculoarticular complex.[7, 8] However, the passive torque represents the global resistance of the whole passive musculo-articular complex to the motion, and therefore involve several structures (muscles, tendons, joint capsule, skin, nerves, etc.). [9–11] Therefore, this method does not assess the behavior of each hamstring muscle, while these muscles differ from their architectural properties [12–14] that may impact their function [15] and could lead to different levels of stiffness within in the muscle group and predispose to injury. [16]

In order to target one muscle, fascicle lengthening can be measured using ultrasound.[17–19] However, fascicle length alone cannot be used to provide information on muscle tension or stiffness. In addition, fascicle length measurements are feasible using ultrasound on only a few muscles (*gastrocnemius*, *vastus lateralis*) that have an appropriate architecture (i.e., unipennated) and with B-Mode images of sufficient quality. Thus, while hamstrings are very frequently injured with a high rate of re-injuries,[20–22] this muscle group is the focus of much less attention than the triceps surae.[23] To our knowledge, only one study has measured the fascicle length of one hamstring muscle (semimembranosus) during passive stretching.[24] Therefore, while strong anatomical differences between muscles suggest that they are likely to behave differently,[12, 25] the mechanical properties of each hamstring muscle has not yet been reported *in vivo*.

Elastography methods, such as *supersonic shear imaging* (SSI),[26, 27] provide the unique opportunity to assess the localized mechanical properties of muscles. It has been shown that the shear modulus measured using this technique is linearly related to the Young’s modulus of the muscle [28] and to passive tension.[29, 30] Two recent studies applied this technique to the hamstrings.[31, 32] Umegaki et al. [31] showed that static stretching differently altered the shear modulus of *semitendinosus*, *semimembranosus* and *biceps femoris*, strengthening the hypothesis of different behaviors for each muscle. However, while elastography measurement can be applied during the muscle lengthening, both studies performed measurements in static condition. It could be argued that relaxation may affect such static measurement,[33] explaining that stiffness measurements of viscoelastic materials are classically performed during slow lengthening. In addition, the link with flexibility and maximal range of motion (measured during passive slow stretching) [7, 8] seems more direct for measurements performed during lengthening compared to static positions.

Stretching procedures are used daily for therapeutic interventions and athletic conditioning. However, while numerous studies have compared techniques, duration and frequency of stretching efficiency on ROM [1, 6, 34] only a few have compared positions of stretching in the same investigation.[35–38] Since the objective of stretching is mainly to tension muscles, it can be argued that a higher muscle tension will be more efficient to stretch the muscles. Thus, elastography provides an interesting approach to compare muscle shear modulus between positions in order to compare their efficiency. For example, while no muscle crosses both hip and ankle joints, a recent study showed that plantar flexors can tolerate an increase amount of passive tension with the hip extended.[39] Thus, the ability to tension muscles is not only related to muscle-tendon length, but also to the perception of the stretch, and the optimal stretching position to tension the hamstrings remains unknown.

Therefore, the objective of this study was to analyze changes in shear modulus of all hamstring muscles during passive knee extensions performed in various hip positions. Pilot studies showed a high inter individual variability in muscle shear modulus values, this variability was analyzed in details. Since hamstring muscles can be difficult to measure using ultrasound and elastography, a preliminary step was to define the reliability for the SSI measurements of each hamstring muscles during passive stretching.

## Materials and Methods

### Participants

A first group of subjects (n_1_ = 15; 23.5 ± 2.3 years; height 178 ± 5 cm; weight 73.4 ± 5.4 kg) was recruited for the reliability analysis. A second group (n_2_ = 18; 23.8 ± 2.6 years; height 178 ± 6 cm; weight 72.9 ± 8.8 kg) participated in the part focused on hamstrings behavior across hip positions. Subjects could participate in recreational sports, but not in any strength or flexibility training at the time of the study. The protocol was approved by the Institutional Ethics Comity (Nantes Ouest IV, reference: n°CCP MIP-04). Participants were informed of the nature of the study before providing a written informed consent, and the procedures were conducted according to the principles expressed in the Declaration of Helsinki.

### Instrumentation

#### Ergometer

**Fig 1:**
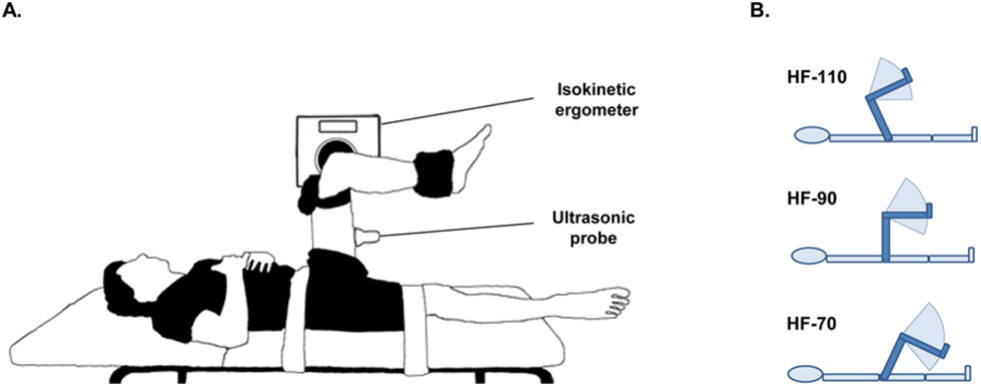
Schematic representation of the experimental setup. (A) Positioning of the subject in the reference position of the knee (90°) at a mid-flexed hip position. (B) Overview of the three stretches with range of motion: high-flexed hip position (HF-110), mid-flexed hip position (HF-90) and low-flexed hip position (HF-70).

A Biodex system 3 Pro (Biodex medical, Shirley, USA) isokinetic ergometer was used to apply passive knee extensions (PKE) and measure knee angle while subjects were lying supine on an examination table (Fig 1A). Pelvis, the right thigh and ankle were fastened using straps. The hip flexion angle was set by inclinometry (Silverline Tools Ltd, ref.250471, Lufton, U.K.). The input axis of the dynamometer was aligned with the presumed axis of rotation of the knee joint. A knee angle of 90° from full extension was our reference and 110° of flexion was defined as the starting knee angle (Fig 1B).

#### Surface electromyographic (sEMG) activity

To ensure that muscles were relaxed during stretching, sEMG activities of *semitendinosus* and *biceps femoris* muscles were measured according to SENIAM recommendations [40] with hydrogel adhesive surface electrodes (Kendall^TM^ 100 foam-series, Covidien, Mansfield, USA). The sEMG activity and passive torque were monitored in real time by both investigators and subject using a feed-back on the computer screen during the trials. The sensor gain was adjusted to ensure stretching procedures were passive (no increase of baseline). If not, procedure was cancelled and reinitiated after a minute of rest. The sEMG data was recorded at 10 kHz using a PowerLab 16/35 data acquisition device (ADInstruments Inc., Colorado Springs, U.S.A.).

#### Muscle shear modulus measurements

An Aixplorer ultrasonic scanner (Supersonic Imagine, v. 6.1, Aix-en-Provence, France), coupled to a linear transducer array (2–10 MHz, SL10-2, Supersonic Imagine, Aix-en-Provence, France) was used in SSI mode (musculoskeletal preset). The SSI technique was previously described in detail.[26, 27, 41] A pushing beam is created in order to generate the propagation of a shear wave within the muscle. Note that the shear wave propagates in 3D, but shear waves velocity is measured in 2D along the principle direction of the probe.[42] Then, ultrafast ultrasound sequences are used to measure the shear wave velocity (*Vs*) using a time-of-flight algorithm [41] in each pixel of the map. Assuming a linear elastic behavior,[26, 27] a shear modulus (*μ*) is calculated using *Vs* as follows:

$$\mu ={\rho Vs}^{2}\;\text{where}\;\rho \;\text{is the muscle mass density}\;(1000\;\text{kg}/{\text{m}}^{3}).$$ (1)

The probe was orientated parallel to the main axis of the muscle to measure the shear modulus along the muscle lengthening direction.[43] Appropriate alignment was achieved when fascicles could be seen without interruption across the image during the stretch.[30, 44] The timing of each shear modulus measurement (i.e., 1 Hz) was recorded by a Powerlab 16/35 data acquisition device (ADInstruments Inc., Colorado Springs, U.S.A.) as a triggering pulse from the ultrasound scanner. Given that the pulse lasts less than 50 ms,[26] it enabled a perfectly synchronization with the knee angles and sEMG signals. Knee angles and sEMG amplitudes were calculated by LabChart software (v.8.0, ADInstruments Inc., Colorado Springs, U.S.A.).

### Protocol

**Fig 2:**
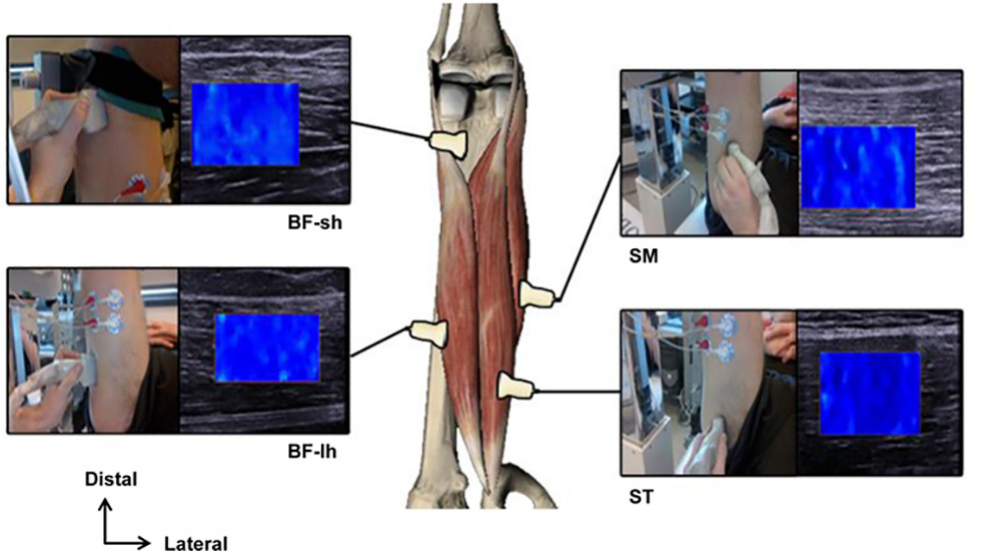
Probe location on each hamstring muscle.

The protocol was designed to measure the shear modulus for each hamstring muscle during a PKE: *semitendinosus* (*ST*), *semimembranosus* (*SM*), *biceps femoris* long-head (*BF-lh*) and *biceps femoris* short-head (*BF-sh*). Based on several pilots and ultrasound of key hamstrings locations, we determined reproducible locations (Fig 2) for maintaining a shear wave map in the targeted muscle during movement.[12, 25] Briefly, we scanned *ST* proximally before the tendinous inscription dividing this muscle into 2 parts.[25] For *SM* and *BF-lh*, the probe was placed after the musculotendinous junction, close to mid-thigh were the muscle belly was sufficiently developed. Finally *BF-sh* was explored distally deeper than *BF-lh*. Based on pilots, we chose a hand-held technique which was more suitable for hamstrings scanning compared to a cast use as done for *gastrocnemius medialis*.[29, 33, 39] The examiner was trained in the use of the Aixplorer machine for hamstring scanning. This involved maintenance of minimal transducer pressure on skin, and the use of caution to not record noise appearance (e.g., flashes or saturation in the region of interest (ROI)).

The first part of the study (n_1_ = 15) was performed to assess the intra-day reliability (i.e., repeatability) of measurements with the hip joint set at 90°. Subjects were asked to stay as relaxed as possible. First, the maximal ROM was assessed as the joint angle corresponding to the maximum perceived and tolerated hamstrings PKE. The stretch ROM was set from 110° of knee flexion until 80% of the maximum ROM, to reduce a possible increase in sEMG at the end of the stretching procedure.[45] Second, five cyclic passive stretches (10°/sec) were performed for conditioning purposes. Third, after a minute of rest, a shear modulus measurement was recorded during a PKE (1°/sec) for each muscle in a randomized order (3 trials per muscle), with one minute of rest set between each repetition. By the end, subjects were positioned with the right hip and knee flexed at 90° (see Fig 1), and they were asked to perform three isometric maximum voluntary contractions (MVC) in knee flexion to normalize the root mean square of the sEMG signals (sEMG-RMS).

The second part of the experiment (n_2_ = 18) was performed to assess the hamstring shear modulus among 3 stretching positions. This set of experiments was performed after the processing data from the reliability study. For each hip angle (70°, 90° and 110° of flexion; HF-70, HF-90 and HF-110 respectively; shown in Fig 1B as tested in randomized order), the same procedure was used. Initially the maximal PKE ROM was measured. The stretch ROM was also set from 110° knee flexion until 80% of the maximal ROM for the considered hip angle. Next, five repetitions (10°/sec) were performed from 110° of knee flexion until 80 of the maximal ROM. Finally, one trial per muscle (tested in a randomized order) was performed to measure the shear modulus of each hamstring. One minute of rest was set between each trial.

### Data analysis

**Fig 3:**
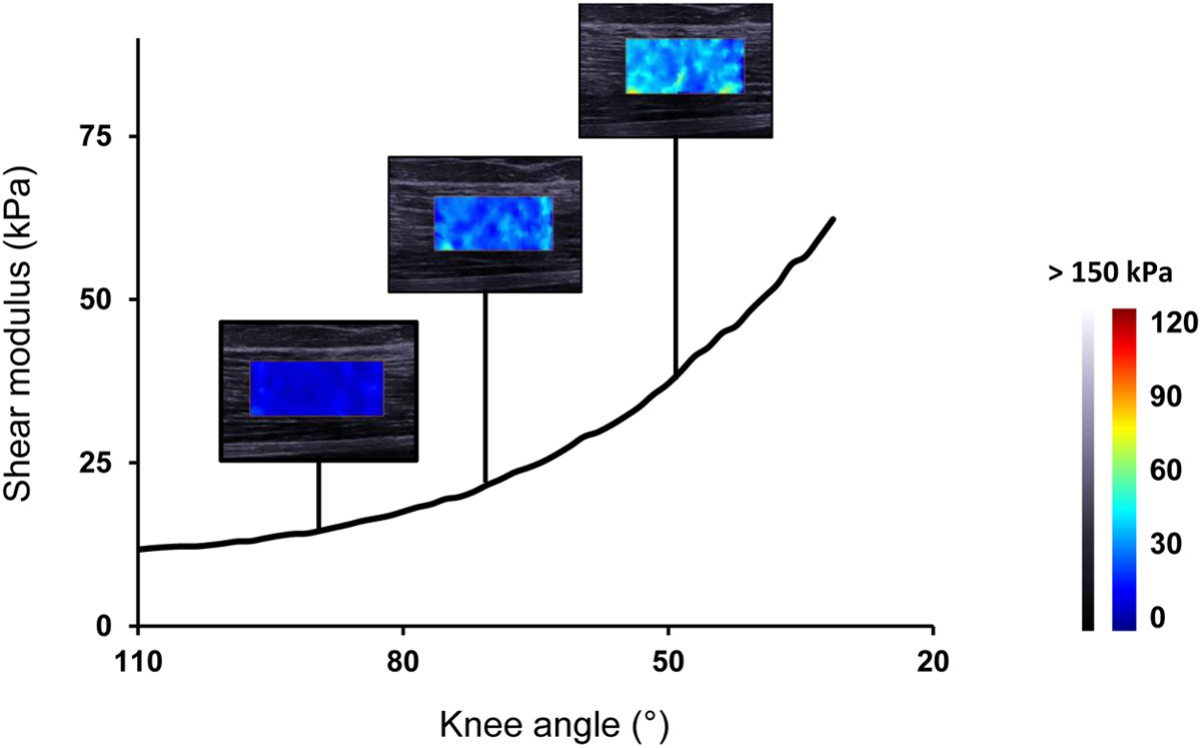
A typical shear modulus-lengthening relationship for *biceps femoris* long-head during the passive knee extension. The colored region represents the map of shear elastic modulus, during the passive stretching procedure at 110° of hip flexion.

All data were processed using standardized programs computed with Matlab^®^ (R2013a, The MathWorks Inc., Natick, USA). Mean shear modulus values were obtained on the largest ROI as possible, without artifacts and aponeurosis. The average areas of the ROIs used were 174.9 mm^2^ for *ST*, 196.2 mm^2^ for *SM*, 194,1 mm^2^ for *BF-lh* and 97.4 mm^2^ for *BF-sh*. For that purpose, data were exported in ‘mp4’ format and transformed to a sequence of ‘jpeg’ images. Image processing then converted the resulting colored map into shear modulus values. Thus, a modulus-length relationship was obtained for each trial (Fig 3 for a typical example). The sEMG-RMS were calculated for each shear modulus measurement and each muscle (1 Hz) and normalized to the maximal values reached during MVC. Normalized sEMG-RMS values higher than 2% where excluded from further consideration.

### Statistics

Data was tested for normality by Shapiro-Wilk test (p-value>0.05) using STATISTICA^®^ software (v.10; StatSoft Inc., Tulsa, USA). Descriptive statistics were reported using the mean and the standard deviation (mean ± SD).

Intra-day reliability was analyzed as proposed by Hopkins,[46] with the two-way random intraclass correlation coefficient (ICC), the standard error in measurement (SEM), and the coefficient of variation (CV) in three angles: 30%, 60%, and 90% of the stretch ROM. Pilot results showed high inter-individual variability in shear measurements. This variability was quantified using the ratio SD/mean (expressed in %) at the maximal common knee angle in HF-110.

For each trial, shear modulus was interpolated at the knee angle corresponding to 30%, 60%, and 90% of the stretch ROM in HF-110 (89.7°, 70.5°, and 50.3°, respectively). We performed one 3x3 repeated measures analysis of variance (ANOVA) to determine muscle, knee angle, and hip position effects on shear modulus. A second ANOVA was performed to determine hip position (70°, 90° and 110° of flexion) and muscle effects on shear modulus at the end of stretch ROM (i.e., 80% of maximal ROM for each hip angle, corresponding to the same subjects’ perception of stretching). Bonferroni post hoc analyses were conducted when appropriate. The level of significance was set at p<0.05.

## Results

One subject was excluded of reliability study and from the second part (effects of hip, knee and muscle) because of painful paresthesias during the experiment. Another participant was excluded from the reliability analysis because of an unsubstantial amount data recorded from the *BF-sh* muscle during the experiment. Therefore, the results below were obtained on 13 subjects (reliability) and 17 subjects (effects of hip, knee and muscle), respectively.

Results of the reliability study are reported in Table 1. The within session repeatability reveals a SEM of shear modulus ranging from 1.1 to 4.2 kPa, corresponding to a CV consistently lower than 15% (8.6%- 13.4%) for *ST*, *SM* and *BF-lh* muscles. However the values are higher for *BF-sh* (CV between 20.3% and 44.9%). In addition, the ICC ranges from 0.71 (*SM*, 30% of the stretch) to 0.94 (*BF-sh*, 30% of the stretch).

**Table 1:** Repeatability of shear modulus measurements, for the HF-90 position.

Percent of ROM		30%	60%	90%
**ST**	**Shear elastic modulus (kPa)**	9.7 (2.5)	16.8 (3.7)	29.5 (7.8)
	**SEM (kPa)**	1.3	1.4	3.4
	**CV (%)**	13.4	8.9	13.1
	**ICC**	0.78	0.89	0.84
**SM**	**Shear elastic modulus (kPa)**	10.4 (1.9)	20.7 (4.7)	40.5 (8.5)
	**SEM (kPa)**	1.1	2.5	4.0
	**CV (%)**	11.2	11.1	10.3
	**ICC**	0.71	0.76	0.81
**BF-lh**	**Shear elastic modulus (kPa)**	13.2 (3.6)	22.6 (6.3)	37.5 (8.8)
	**SEM (kPa)**	1.1	1.8	4.2
	**CV (%)**	10.4	8.6	13.3
	**ICC**	0.92	0.93	0.81
**BF-sh**	**Shear elastic modulus (kPa)**	11.3 (12.6)	17.2 (11.4)	35.8 (21.6)
	**SEM (kPa)**	3.5	5.0	10.6
	**CV (%)**	20.3	36.1	44.9
	**ICC**	0.94	0.84	0.79

Data are shown as mean values (± standard deviation)

Abbreviations: ROM: range of motion, ST: semitendinosus, SM: semimembranosus, BF-lh: biceps femoris long head, SEM: standard error in measurement, CV: coefficient of variation of the measurement, ICC: Interclass correlation coefficient factor

**Fig 4:**
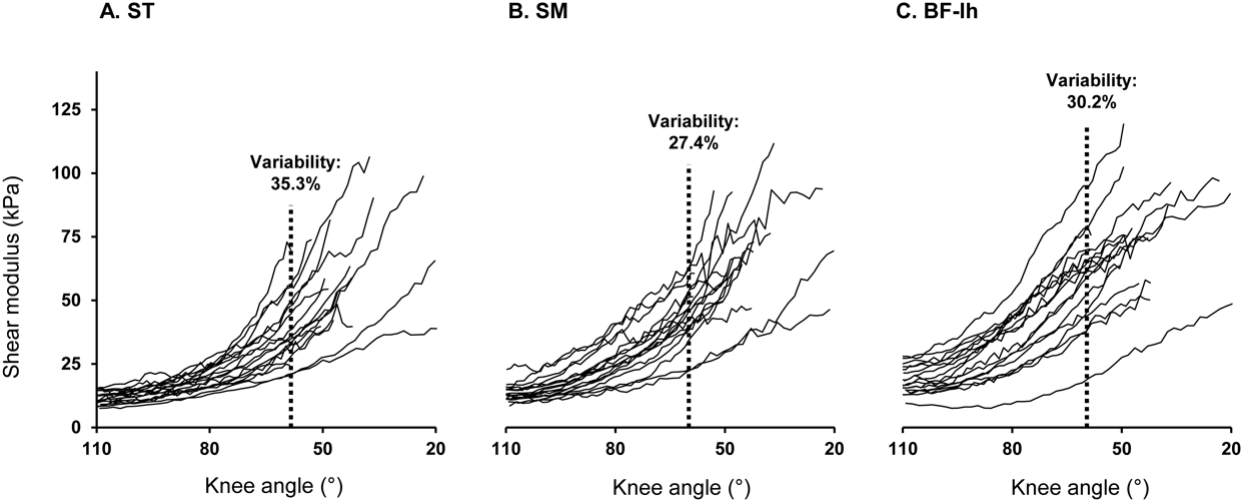
Shear modulus-lengthening relationships obtained for each hamstring muscle from the study participants. The variability was quantified using the ratio SD/mean (in %), expressed at the maximal common knee angle in HF-110 for (A) *semitendinosus (ST)*, (B) *semimembranosus (SM)*, (C) *biceps-femoris* long head (*BF-lh*).

The inter-individual variability of shear modulus is shown on Fig 4. The modulus variation of values between participants was 35.3% for *ST*, 27.4% for *SM* and 30.2% for *BF-lh* at the maximal common knee angle (58°).

**Fig 5:**
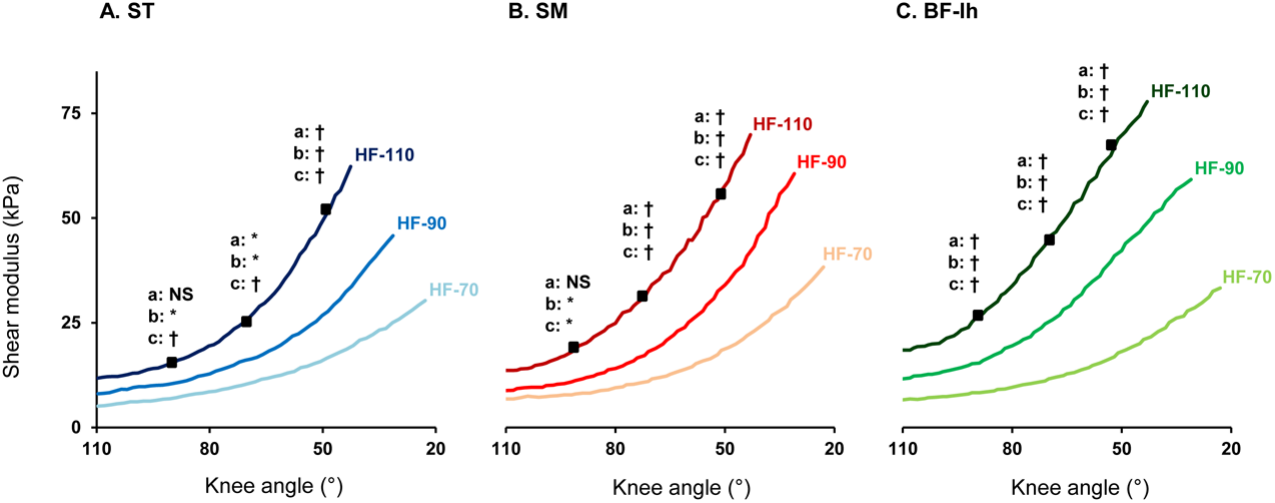
Comparison of hamstring responses in three positions of passive knee extensions, reported by muscle. Shear modulus-lengthening relationships shown for (A) *semitendinosus (ST)*, (B) *semimembranosus (SM)*, (C) *biceps-femoris* long head (*BF-lh*), with interactions for the three same knee angles: a: between 70° and 90° of hip flexion; b: between 90° and 110° of hip flexion; c: between 70° and 110° of hip flexion. Levels of significance: * p<0.05; † p<0.001; NS: no significant at p<0.05

ANOVA revealed three main effects: knee angle (p<0.001), hip angle (p<0.001) and muscle (p<0.001). In addition, significant interactions between ‘muscle x knee angle’ (p<0.001), ‘hip x muscle’ (p<0.001), ‘knee x hip’ (p<0.001) and ‘hip x muscle x angle’ (p<0.05) were found. As expected, a notable increase in shear modulus was observed when the knee was extended (Fig 2 for a typical example). Shear modulus values were, as presumed, higher in the three knee angles when the hip was flexed (p<0.001) for the three tested angles (black squares in Fig 5) with mean values of 12.3±5.7 kPa at HF-70, 22.4±12.8 kPa at HF-90, and 38.5±21.3 kPa at HF-110. Averaged shear modulus-length relationships are shown for each muscle in Fig 5 (Fig 5A for *ST*, Fig 5B for *SM* and Fig 5C for *BF-lh*).

**Fig 6:**
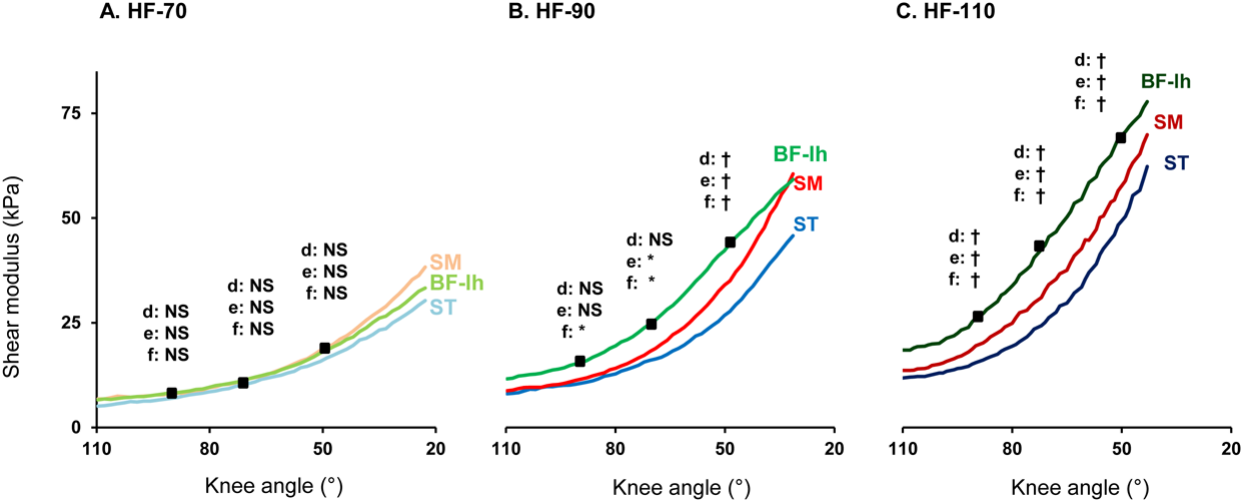
Comparison of hamstrings responses in the three positions of passive knee extension, reported by hip positioning. Shear modulus-lengthening relationships shown for (A) 70° of hip flexion, (B) 90° of hip flexion (C) 110° of hip flexion, with interactions for the 3 same knee angles: d: between *ST* and *SM*; e: between *SM* and *BF-lh*; f: between *ST* and *BF-lh*. Levels of significance: * p<0.05; † p<0.001; NS: no significant at p<0.05

Moreover, our data indicate significant differences between muscles (p<0.05) with higher shear modulus values for the *BF-lh* (32.6±21.6 kPa) compared to the *SM* (27.8± 17.2 kPa), and the values for the *SM* higher than the *ST* (22.9±15.6 kPa). The averaged shear modulus-lengthening relationships across muscles are shown in Fig 6. For HF-70, no significant differences (p>0.05) were found between muscles responses for the three knee angles (black squares in Fig 6A). For HF-90 significant differences between the three muscles are reported in the last knee angle (p<0.001, Fig 6B). For the high-flexed hip position (HF-110), significant differences in shear modulus were found between the three muscles at the three knee angles (p<0.001, Fig 6C).

The two way ANOVA for the maximal shear modulus reached at the end of the stretch ROM, showed significant main effects of hip angle (p<0.001), muscle (p<0.05), and a significant ‘hip x muscle’ interaction (p<0.001). Interestingly, at the end of the ROM when comparing maximal shear modulus measurements corresponding to the same maximal perception of stretch (*i*.*e*., 80% of the maximal PKE ROM for each hip angle), the highest values were always reached at HF-110 (62.3±22.0 kPa for *ST*, 69.9±17.8 kPa for *SM*, and 77.8±20.5 kPa for *BF-lh*) by comparison to HF-70 (30.3±11.2 kPa for *ST*, 38.3±10.0 kPa for *SM*, and 33.3±9.8kPa for *BF-lh*) and HF-90 (45.8±18.7 kPa for *ST*, 60.6±17.0 kPa for *SM*, and 59.2±15.2 kPa for *BF-lh*).

## Discussion

The present study was designed to assess hamstrings response during passive stretching by measuring shear modulus using SSI elastography. Based on reliable ultrasonic key locations of the muscles (see Fig 2) [12, 25] we investigated the whole group during PKE performed in three different positions.

Considering that ICC and threshold are often used in the literature, reliability was good for all the conditions tested in the present study (Table 1).[47] However, the ICC is by definition dependent on a heterogeneous set of values. For example, if a between-subject variability is low compared to the within-subject variability, the ICC tends to be low as demonstrated by Weir.[48] Thus, ICC should not be reported alone, but with an absolute estimation of error such as SEM and CV. [46, 48] Therefore, the within-session intra-operator reliability was considered as acceptable for *ST*, *SM*, and *BF-lh* muscles (SEM and CV lower than 4.2 kPa and 15%, respectively). This was not the case for the *BF-sh* (SEM and CV higher than 10 kPa and 40%). Although the examiner was highly trained by pilots during the previous months, CV values reported in the present study were slightly higher than in the literature (e.g., 1.92% in *tibialis anterior* [49], 5.6% for *biceps brachii* and *vastus lateralis* [50] 7.5% in *gastrocnemius medialis* [29]). There are three main reasons that could explain this finding. First, hamstrings are more difficult to analyze using ultrasound, due to a complex organization (*e*.*g*., intra-muscular fibrous composition) [12, 25] compared to clearly structured muscles such as *gastrocnemius*, *quadriceps*, *biceps brachii*, and *tibialis anterior* muscles. Secondly and linked with the previous point, we performed hand-held measurements that may increase the variation of measurement, compared to a cast-positioned transducer on the muscle belly. Thirdly, we decided to perform measurements during passive slow stretching (i.e., dynamic condition). Static experiments would be result in simplified scanning.[23] However, it would imply stress relaxation [33] and conventional methods of material testing involves characterization at a slow strain rate.[7–9] Thus, we decided to perform measurements during the stretching even if it implied an experimental lower reliability. In addition, *BF-sh* scanning was made on the distal part of the thigh close to the isokinetic ergometer (Fig 2) and corresponded to a transducer placement targeting the deepest muscle with shear waves propagating under a superficial stiff material (tendon of *BF-lh*). Both reasons could explain the low reliability obtained for this muscle (CV between 20.3% and 44.9%). Future will need to assess these problem in order to assess the particular influence of this mono-articular muscle on the stress distribution of the group.

Since *SM* and *BF-sh* activities were not measured using sEMG, it was not possible to check that these muscles were passive. However, no recommendation were made in the SENIAM project for these muscles,[40] probably because they are quite difficult to measure. Based on two muscles and a careful check of the torque-angle curve, it was assumed that the four hamstrings were passive, but it remains a limitation of the present study.

Our results demonstrate that the shear modulus of each measured hamstring is increased during the PKE at a given knee angle for more flexed hip position (Figs 5 & 6, p<0.001). This result is in accordance with the biarticular behaviors of these muscles. Thus, the present study confirms that the shear modulus can be used to estimate changes in passive muscle tension or muscle-tendon length of biarticular muscles like the hamstrings.[29, 30]

In the present study, as done in most of research and clinical settings, subject sensation was used to end a stretch procedure.[9] Thus, if the perception of the stretch is directly linked to the passive muscle tension (estimated using the shear modulus), peak shear modulus values reached at the end of the stretch ROM would have been identical for each hip position. However, our results clearly show that this is not the case, with a peak shear modulus that significantly increased when the hip was flexed (Fig 5, p<0.001). In other words, the amount of passive muscle tension locally reached was different at the end of the PKE for the three investigated stretch positions despite a similar perception. Therefore, these results reveal a lower ability of the hamstring muscle to tolerate stress in the low-flexed hip PKE (HF-70 and HF-90) compared to HF-110. The exact mechanism is still unknown, but it shows that tissues other than muscle play a definite role in the stress tolerance for ending the stretch, particularly in low-flexed hip situations where local muscle passive tension stay low.[9–11] These results agree with those of previous studies performed on the ankle [39, 51] that suggest the potential role of nerves or fascia in the limitation of the ROM in some joint configurations. Therefore, when the aim is to focus on the muscle-tendon component, our results suggest flexion of the hip as much as possible to increase the efficiency of stretching procedures or hamstring flexibility measurements.

Results of the present study also showed clear differences between muscles shear modulus during a stretch. It is in accordance with a recent study that performed measurements in one static position.[31] However, *BF-lg* was found to be the stiffest muscle in the present study, while Umegaki et al. [31] showed that the shear elastic modulus was higher for the *SM* muscle. The discrepancy can not only be explained by differences in cross-sectional area, specific tension, degree of anisotropy and passive force-length relationship between muscles,[52] but also by methodological differences between the studies. Specifically, two main methodological differences were emphasized here and might partly explain the differences in results. First, Umegaki et al. [31] performed measurements at static conditions for one knee angle, while we performed measurements on the whole stretch ROM during PKE. Due to stress relaxation, the shear modulus value clearly decrease during time for a static stretching protocol.[33] Therefore, shear modulus measurements seem more appropriate during slow dynamic protocols as performed in the present study. Second, Umegaki et al. [31] placed the probe at the midpoint from the greater trochanter to the femoral epicondyle (medial or lateral). Structural organization of hamstring muscles was shown to be very variable within a muscle and between subjects.[12–14] Thus, we preferred to set locations according to hamstrings ultrasound key locations [12, 25] (described above in protocol section, and illustrated in Fig 2) in order to investigate a constant area in each muscle. Therefore probe locations varied greatly between studies, and it remains unclear how placement can influence shear modulus measurements.

In elastography studies a constant muscle density is classically assumed [26] (1000 kg/m^3^ in the present study). Further elastography studies are required to better understand and clarify the spatial variability of shear measurements for different muscles of a same muscle group. In addition, the shear modulus was performed in only one location for each muscle. It remains to be determined whether shear modulus varies within one muscle during passive stretching.

Interestingly, results of the present study showed that the more the hip is flexed, the more the shear modulus differed between *BF-lh*, *SM*, and *ST* with the highest increase for *BF-lh* (Fig 6). In addition, the slack angle, defined as the angle beyond which shear modulus is increased,[29, 53] was similar between the muscles. Below the presumed slack angle, no differences between muscles in resting shear modulus were found (Fig 6A). Therefore, these results suggest that hamstring muscles display different mechanical behaviors during the PKE, also depending on the hip joint position. This could be related to differences in cross-sectional area, specific tension, degree of anisotropy, and parameters that govern the normalized passive muscle force-length relationship between muscles.[52] Particularly, the shear modulus could be influenced by the in-series organization of muscle and tendon. For instance, *BF* and *SM* display the longest length of musculotendinous junctions,[12, 25] and it may partly explain differences in values reported in the present study.

Interestingly, the shear modulus values perfectly match the prevalence of hamstring injuries.[20–22] A recent study of Hallén & Ekstrand [54] reported 83% of hamstring injuries occurring for *BF* (12% for *SM* and 5% to *ST*) in a 12 year European-level professional football teams follow. Our results would corroborate that muscle mechanical properties, may be involved in the risk of injury.[16, 55] In addition, a high inter-individual variability was found for shear modulus values at the same knee angle (35.3% for *ST*, 27.4% for *SM* and 30.2% for *BF-lh*). This heterogeneity of stress tolerance/behaviors should also be taken in account for hamstring flexibility examinations. Considering the prevalence in hamstring strain injuries and lack of progress in solving these type of injuries,[20–22, 56] further elastography studies are required and should analyze the relevance of the shear modulus measurement in the injury prediction or in the evaluation of acute and chronic effects of stretching programs.

## Conclusion

This study quantified the shear modulus of hamstring muscles during a passive knee extension, and reported mechanical measurements at a more local scale than usually done by conventional methodologies. The results highlight different stress tolerances to stretching, depending on hip position, and that hip flexion level appears to be fundamental tied to efficient stretching in the muscle-tendon component during a passive knee extension. Moreover, even if hamstrings tend to have a similar slack angle, *BF* (long-head) shear modulus is higher than the other muscles. While this study was performed with healthy subjects, our methodology would also be relevant for the clinical evaluation of disorders involving these muscles. Finally future studies will help to provide an evidence based practice of hamstring extensibility examinations and stretching procedures.

## Supporting Information

S1 File Elastography data for all the participants (S1 to S18) in the various positions. (HF-70: hip flexed at 70°, HF-90: hip flexed at 90°, HF-110: hip flexed at 110).
(XLSX)

S2 File Elastography data for the reliability analysis for all the participants (S1 to S15) in the various positions. (HF-70: hip flexed at 70°, HF-90: hip flexed at 90°, HF-110: hip flexed at 110).
(XLSX)
